# Supplementary material for: Splice-Junction-Based Mapping of Alternative Isoforms in the Human Proteome
Source: Cell Rep. Author manuscript; Available in PMC 2020 Jan 15. (PMC6961840; doi:10.1016/j.celrep.2019.11.026)
Supplement: 2 [file NIHMS1546469-supplement-2.zip › DF1/PXD006675/CardiacFibroblast/CardiacFibroblast_15_JARID2_VPSIIQK.pdf]

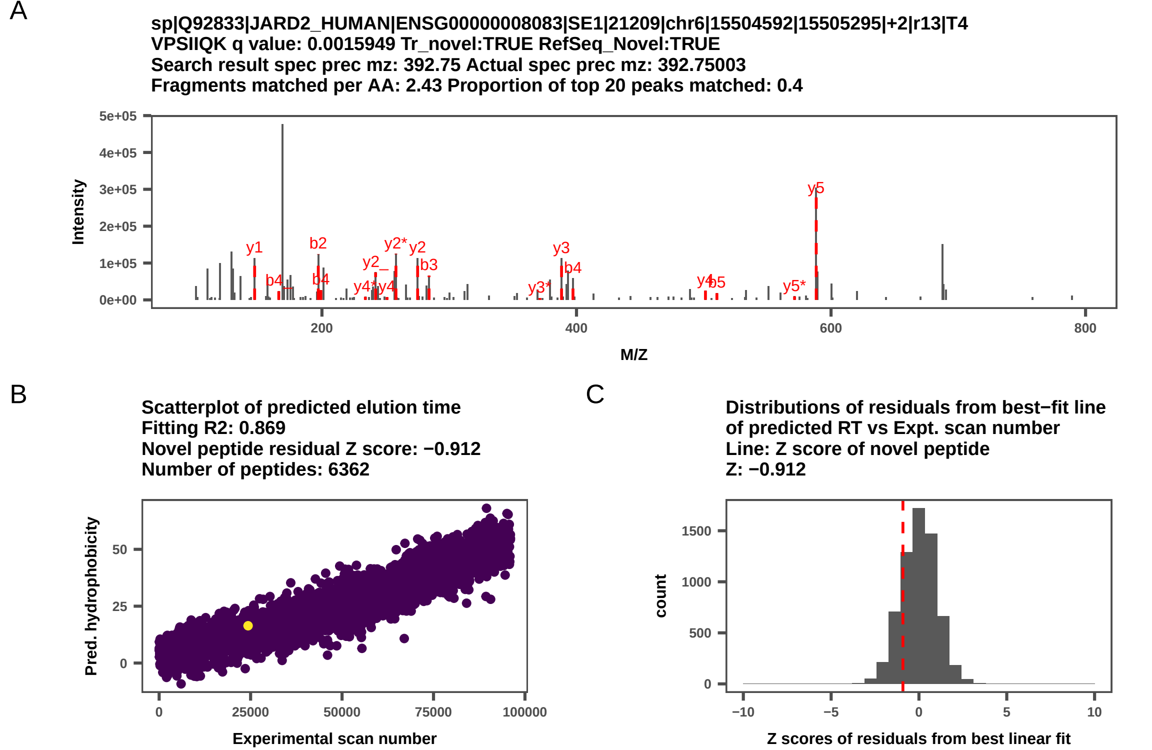
A
sp|Q92833|JARD2_HUMAN|ENSG00000008083|SE1|21209|chr6|15504592|15505295|+2|r13|T4
VPSIIQK q value: 0.0015949 Tr_novel:TRUE RefSeq_Novel:TRUE
Search result spec prec mz: 392.75 Actual spec prec mz: 392.75003
Fragments matched per AA: 2.43 Proportion of top 20 peaks matched: 0.4
Intensity
M/Z
B
Scatterplot of predicted elution time
Fitting R2: 0.869
Novel peptide residual Z score: −0.912
Number of peptides: 6362
Pred. hydrophobicity
Experimental scan number
C
Distributions of residuals from best−fit line
of predicted RT vs Expt. scan number
Line: Z score of novel peptide
Z: −0.912
count
Z scores of residuals from best linear fit
